# An Efficient Multilevel Probabilistic Model for Abnormal Traffic Detection in Wireless Sensor Networks

**DOI:** 10.3390/s22020410

**Published:** 2022-01-06

**Authors:** Muhammad Altaf Khan, Moustafa M. Nasralla, Muhammad Muneer Umar, Ghani-Ur-Rehman, Shafiullah Khan, Nikumani Choudhury

**Affiliations:** 1 Institute of Computing, Kohat University of Science & Technology, Kohat 26000, Pakistan; dr.altaf@kust.edu.pk (M.A.K.); muneer.umar@kust.edu.pk (M.M.U.); skhan@kust.edu.pk (S.K.); 2 Department of Communications and Networks Engineering, Prince Sultan University, Riyadh 11633, Saudi Arabia; 3 Department of Computer Science and Bioinformatics, Khushal Khan Khattak University, Karak 27000, Pakistan; ghani.rehman@kkkuk.edu.pk; 4 Department of Computer Science and Information Systems, Birla Institute of Technology & Science, Pilani, Hyderabad 500037, India; nikumani@hyderabad.bits-pilani.ac.in

**Keywords:** WSNs, security, DDoS, flash crowd, Bayesian model

## Abstract

Wireless sensor networks (WSNs) are low-cost, special-purpose networks introduced to resolve various daily life domestic, industrial, and strategic problems. These networks are deployed in such places where the repairments, in most cases, become difficult. The nodes in WSNs, due to their vulnerable nature, are always prone to various potential threats. The deployed environment of WSNs is noncentral, unattended, and administrativeless; therefore, malicious attacks such as distributed denial of service (DDoS) attacks can easily be commenced by the attackers. Most of the DDoS detection systems rely on the analysis of the flow of traffic, ultimately with a conclusion that high traffic may be due to the DDoS attack. On the other hand, legitimate users may produce a larger amount of traffic known, as the flash crowd (FC). Both DDOS and FC are considered abnormal traffic in communication networks. The detection of such abnormal traffic and then separation of DDoS attacks from FC is also a focused challenge. This paper introduces a novel mechanism based on a Bayesian model to detect abnormal data traffic and discriminate DDoS attacks from FC in it. The simulation results prove the effectiveness of the proposed mechanism, compared with the existing systems.

## 1. Introduction

During the last few decades, the sensor-based ad hoc network known as wireless sensor network (WSN) has become popular in various fields. By deploying WSNs in various fields, many real-world problems have been inexpensively and easily solved [1,2]. These networks are composed of thousands of interconnected low-cost nodes. Every node has limited computational and communication power with some sensing capabilities [3]. The deployed sensor nodes gather targeted information from the area being monitored. The collected data is then forwarded via intermediate sensor nodes to the base station (BS) [4].

The primary objective of the routing protocols used in WSNs is to enable nodes to send their data to the BS. Most of the routing protocols do not provide any mechanism against malicious attacks. Due to the administrativeless, unattended environment and insecure routing protocols, WSNs are vulnerable to various types of security attacks. These attacks include eavesdropping, sinkhole, traffic analysis, camouflage, blackhole, and denial of services (DoS) attacks.

Among the common attacks, the DoS attack, which is carried out on the network layer, is very popular. In this attack, the attacker tries to stop the victim’s ability to use its bandwidth or respond to any request. DoS attack is obtained by transmitting bulky requests to the victim in a short period of time. Upon receiving such requests in bulk, the victim node becomes incapable of responding or processing the requests. During this period, the legitimate request would also not be entertained by the victim node [5,6]. While having such a scenario, the entire network’s performance is highly affected. The most severe case of the DoS attack is the distributed DoS (DDoS) attack.

DDoS attacks are carried by a group of attackers, distributed in the network at various positions. The distributed attackers combinedly launch a DoS attack against a single node or the entire WSN. Usually, DDoS attacks are launched through some compromised nodes with an objective to disturb the functionality of some nodes or the entire network [7]. The detection of DDoS attacks obtains more importance when they happen simultaneously with flash crowd (FC) traffic [8]. FC is actually the heavy traffic generated by legitimate users. An FC event occurs due to the unexpected increase in traffic with some special events such as an emergency alarm for severe heat situations. FC tries to access the server simultaneously, resulting in unexpected overflowing [9]. As attackers use different techniques and tools to generate DDoS attacks, it is very difficult to detect DDoS attacks. Both DDoS and FC are forms of abnormal traffic. In both cases, the target nodes during communication obtain the bulk of requests from various sources. In some special scenarios, the FC affects the network performance in the same ways as the DDoS. However, the primary intention of the nodes generating abnormal traffic is considered. The abnormal traffic with no malicious intention cannot be treated as a DDoS attack. The detection and identification of abnormal traffic are very essential and are a challenge in many wireless ad hoc networks.

Conversely, in DDoS attacks, the illegal users transmit a large number of request packets with the purpose to stop the server from providing services. UDP flooding, HTTP flooding, and TCP SYN flooding are well-known examples of DoS attacks. DDoS attacks are launched through botnets; the group of compromised nodes is called a botnet. The bots in a single botnet follow command directions from a botmaster. To initiate an attack session, the botmaster gives instruction commands to all its concerned bots, resulting in the target victim shutting down due to a large number of packets. Different detection mechanisms can be applied to overcome a DDoS attack [10]. DDoS attacks and FC possess many similar features and behaviors. In both cases, single or multiple nodes are overloaded, and the network connection is congested due to heavy incoming traffic. In addition, some differences exist between DDoS attacks and FC, i.e., variations in the incoming traffic speeds, source IP addresses distributions, and their access intents. The behavioral differences between FC traffic and DDoS attack must be analyzed to differentiate between them [11,12].

The WSNs are highly vulnerable to DDoS attacks due to the diverse nature of these networks. The tiny sensor nodes in such types of networks cannot be simply programmed with bulky code or equipped with additional components to overcome DDoS attacks. The sensor nodes are deployed in an unattended manner in the target fields. Therefore, the attacker nodes can easily be deployed, and these intruder nodes can easily overhear the broadcast transmission among the wireless sensor nodes. The nodes are unattended; therefore, these nodes cannot be manually checked for ensuring that no unknown nodes are added or replaced with some existing nodes in the network. Moreover, both the DDoS and FC have similar appearances; therefore, identification of these is also a challenging task. In WSNs, the presence of DDoS attacks can cause a variety of issues. The following is a breakdown of the issues:

(a) The buffer space of a BS has been filled due to a large volume of traffic, and communication between the BS and normal nodes has come to a halt.
(b) The fraction of often-missed packets is particularly high due to network connection overload.
(c) Due to network congestion, only a few packets from regular nodes make it to the target BS, but not in a timely manner. As a result, network throughput suffers.
(d) DDoS attack is a form of abnormal traffic. The abnormal traffic can also be FC. A mechanism merely blocking the abnormal traffic, assuming it is a DDoS attack, may lose some important and legitimate users, too.
(e) A sophisticated and efficient mechanism for discriminating FC from DDoS is always required to prevent sensor nodes from malicious attacks but not the FC-based abnormal traffic.

In this paper, an intelligent detection mechanism based on the Bayesian model is introduced to detect and discriminate DDoS attacks from FC traffic. To make the distinction more efficient, different characteristics of DDoS attacks and FC traffic are analyzed. A few basic parameters are recognized that are used for discriminating DDoS attacks from FC. These parameters are the number of packets generated, or inflow, the packet payload size, and packet interarrival time variances. The detection and discrimination are made by using these three aforementioned parameters. The detection mechanism mainly focuses on the received packets and traffic; therefore, it is deployed at the BS. The main overhead is carried by the resourceful station. The nodes do not need any additional operations while performing their regular tasks. The proposed mechanism does not affect the working condition of the sensor nodes, as the main operations regarding the detection and discrimination of FC from DDoS attack are performed at the BS. At the end, their results are provided to show the effectiveness of our proposed work.

The rest of this paper is organized as follows: Section 2 discusses the background and related work. Section 3 presents the proposed mechanism. In Section 4 and Section 5, results based on simulations and by using real datasets are given, respectively. Finally, the paper is concluded in Section 6, and also future guidelines are provided for future research.

## 2. Background and Related Works

A lot of research efforts have been carried out to detect and mitigate DDoS attacks, and various defense techniques have been proposed with different concepts. These attacks mainly target the network layer, transmitting a large number of packets to exhaust the victims’ resources. A wide range of defense techniques has been developed to overcome DDoS attacks.

In a mechanism, proposed in [13], a naïve classifier is used to categorize the incoming traffic into attack or normal traffic. According to the proposed work, the naïve classifier gains an accuracy of up to 99%. Another mechanism, in [14], classifies the incoming data and control packets into the attack and normal types by availing the algorithm of statistical packet classification. For obtaining accuracy, the K-NN algorithm is used to optimize the results generated by the statistical packet classification algorithm. Some authors propose an abnormal event detection in WSN by observing the correlation among various attributes [15]. A Bayesian network is designed based on the dependency model of the observed attributes. Structure learning is used to acquire the dependence structure of observed characteristics, while parameter learning is used to calculate the conditional probability table of each node. The main objective is to distinguish the abnormal events relating to sensor reading. The work has used the Bayesian network in a very good manner. However, the work is targeting a different domain than DDoS and FC discrimination.

A naïve Bayesian classifier-based DDoS attack detection and mitigation mechanism is proposed by Reddy and Thilagam [16]. In this work, an additional node authentication system using RSA-1024 is also used. Five main network parameters are considered. These are packet size, port number, source address, destination address, and jitter. The work mainly focuses on the DDoS attack detection but does not consider the FC traffic.

In [17], an intelligent detection mechanism is proposed which applies the combination of support vector mechanism (SVM) and radial basis function (RBF) to train the proposed model for DDoS attack detection. With this approach, 98.7% accuracy is achieved under some special cases.

In the approach STONE [6], an online anomaly-based attack detection framework is used. This approach has consisted of two major components: the detection control center and the mitigation center. The first component is responsible for anomaly detection, while the latter is used to filter out and block DDoS attacks. While considering the FC versus DDoS distinguishment, there is limited work published. Normally, the existing detection techniques are either heuristic-based defense techniques or statistical-based defense techniques, which depend on header information of IP (internet protocol) packets to separate valid nodes traffic from malicious nodes traffic. Nevertheless, in these techniques, none of them is efficient when FC events occur because the genuine traffic flow and attack flow share similar properties [8]. Therefore, payload size and arrival time-based detection technique is more desirable when DDoS attacks and FC occur concurrently.

A novel mechanism for differentiating DDoS attacks from FC traffic is proposed in [18]. This mechanism is based on entropy variation. While using the entropy variations, a tracing technique is designed to detect the actual sources of the attack. The network throughput, packet delivery ratio, and traceback time are considered to assess the performance of the proposed mechanism. The performance of the system gradually degrades with the increased traffic rates. The authors have not supplied adequate simulation results.

Shui et al. [19], proposed another similar approach in the target domain. The similarities in the data flow are considered in this mechanism. The calculation of flow similarities is based on three defined metrics: Sibson distance, Jeffrey distance, and Hellinger distance. The authors claimed that the Sibson distance discriminates DDoS attack flow from FC more precisely than Jeffrey and Hellinger distances. This mechanism achieves 65% accuracy, which cannot be considered an efficient solution. There are several things to be considered for DDoS and FC discrimination than merely considering the flow similarity.

The authors in [11] consider the differences between the behavior of DDoS attacks and FC traffic to recognize a set of appropriate parameters. These parameters are further utilized to differentiate between the two classes of traffic flows. The selected parameters are limited, and few simple calculations are performed during the entire mechanism. Therefore, the approach does not obtain an adequate level of accuracy.

A probability metrics-based mechanism is proposed by W. Zhou et al. [20]. The experimental scenarios with performed simulation results indicate that their mechanism pointedly surges the sensitivity and declines the false-positive as well as false-negative rates. The proposed mechanism works well in normal situations, but its performance highly worsens with a sharp increase in the attack as well as flow traffic, while considering merely the flow similarity cannot be suitable for discriminating DDoS attacks from FC traffic.

In [21], T. Thapngam et al. examine the correlation between different packet arrival rates by calculating Pearson’s correlation coefficient to differentiate DDoS attacks from FC traffic. The authors observe that there is a greater level of automation in DDoS attacks with a transmission rate that can be predicted, while in the case of FC traffic, the rate of request packets cannot be predictable. The limitations of the proposed approach are that the botmaster can mislead this mechanism by forwarding mimic attack traffic, and they only work for known patterns.

In [22], Thapngam et al. consider the prebuilt programs as the attack tools in botnets. Therefore, due to prebuilt instructional sets, it is assumed that the flow trends in DDoS attacks are similar, and the ratio of this similarity is higher than FC flows. The proposed mechanism calculates the correlation coefficient of flows generated by various suspicious nodes to distinguish FC traffic from DDoS attacks. However, due to the increased number of bots, the performance of the proposed mechanism degrades. In particular situations, the system fails where the bots’ traffic is very high, compared with the normal traffic by legitimate nodes.

J. Gera et al., in [23], propose an approach based on cluster traffic randomness and source address entropy to detect spoofing as well as nonspoofing DDoS attacks and differentiate them from FC. Furthermore, the DDoS attack and flash crowd are classified into their corresponding categories.

A hybrid scheme is proposed by S. Daneshgadeh in [24]. The authors proposed combining the three different approaches, i.e., Shannon entropy, Mahalanobis distance, and kernel online anomaly detection. Shannon entropy is used based on machine learning techniques to recognize uncommon traffic comprising FC traffic and DDoS attacks. Later on, the Mahalanobis distance metric is used to differentiate FC traffic from DDoS attacks. The performance of the proposed hybrid mechanism is evaluated by using simulation for real, normal traffic, FC traffic, and DDoS attacks. The results show that the Mahalanobis distance metric performed efficiently combined with machine learning techniques to identify and efficiently identify FC traffic and DDoS attacks based on false alarm rates and detection rates. The summary of the related articles is given in Table 1.

In the existing research, most of the mechanisms consider the data flow to detect abnormal traffic. Most articles consider the abnormal traffic as the DDoS attack, while the discrimination of DDoS from FC is limited in current literature. Some authors have used mechanisms that may overload the tiny sensor nodes in WSNs. Many have not used any procedure involving the testing and validation based on datasets. The proposed work introduces a novel technique of a probabilistic model based on naïve classification, which not only detects the abnormal traffic but also identifies it as a DDoS or an FC. For validation of the work, two datasets having records for FC and DDoS are also used in the proposed mechanism.

## 3. Proposed Detection Mechanism

The proposed mechanism, DDoDF, is an intelligent FC and DDoS detection system based on a naïve Bayesian model. The main objective is to differentiate DDoS attacks from FC in network traffic. The targeted type of network is WSN. While keeping the limitations and features of WSNs, the proposed mechanism adapted an optimized procedure to avoid any heavy overhead on the network nodes. To make the distinction more efficient, different characteristics of DDoS attacks and FC traffic are analyzed, and a few basic parameters are recognized that are used for discriminating DDoS attacks from FC. The key parameters used are (a) the number of packets generated or inflow, (b) the size of the packet payload, which is being transmitted or received at nodes, and (c) the packet interarrival time variances.

In most of the literature, these three parameters are considered or mentioned for the detection of DDoS attacks in similar networks. Some examples are [2,5,9,12,18,19]. The sensor nodes in WSNs operate on very limited resources, and it is essential not to deploy complex or bulky mechanisms, which may raise the processing and communication overhead, causing the degradation of network performance. The proposed mechanism’s main focus is to check the nature of packets and nodes’ transmission behavior. It is assumed that considering port numbers while having IP addresses may place an additional load on the nodes. Furthermore, the overloaded authentication mechanisms are always discouraged in WSNs.

The first and the most important parameter is the analysis of the amount of packets being received at a particular node(s). The number of packets generated in particular time intervals can be used to identify the abnormality in the traffic. In general cases, a small number of packets is generated from the sensor nodes upon sensing some environmental change, while in the case of DDoS and FC, the nodes generate a large number of packets either for malicious or nonmalicious purposes.

The second parameter considered is the size of the packet payload. The analysis of payload size can be used to differentiate DDoS attacks from FC traffic. It is obvious that DDoS attacks are initiated by using some attack tools or prebuilt programs. These tools usually generate attack packets of the same sizes or same data patterns, while in the case of FC, all the packets are generated from different legitimate users. Each node may require its particular operation to be performed. Therefore, the packet sizes and their patterns will be not similar in general cases.

The last parameter is the interarrival time variance that is used to differentiate DDoS attacks from traffic. In DDoS attacks, the attack packets are generated from some programmed nodes; therefore, it is obvious that the interarrival time between the packets will be constant or less. It is because the nodes frequently burst packets in a particular pattern to consume the victims’ resources. However, the legitimate nodes transmit their data packets in an unknown or random pattern. Therefore, it is obvious that the in-between time-interval for packets arrival will be different.

There are two major phases in the proposed detection system, detection and discrimination of DDoS Attack (DDoDF). These phases are the training phase and the testing phase. The datasets are obtained and used during the training and testing phases. In this work, 70% of data records from each dataset are used to train the proposed system. Upon completion of the training phase, the testing phase starts. In this phase, the trained system is tested on the remaining 30% of the data from the dataset to check the working validity of the proposed system. Various dimension datasets are used to experiment with the proposed work. The datasets of size of around 10,000 input vectors give the best outcomes in the proposed mechanism during training and testing phases. The input vectors in the datasets are categorized as normal, DDoS attack, and FC traffic packets. In the training phase, the system is provided with the incoming input vectors attached with their desired labeled classes, while in the second phase of testing, the provided vectors are not labeled, and the system performance is checked.

The Bayesian model is a key component in the structural design of the proposed system. The Bayesian model is a mathematical probability-based model. This model consists of three classes of probabilities: (a) prior probability, (b) likelihood probability, and (c) posterior probability. The prior probability is merely the number of instances that go to either a DDoS attack or flash class divided by the total number of instances in both classes.

**Figure 1:**
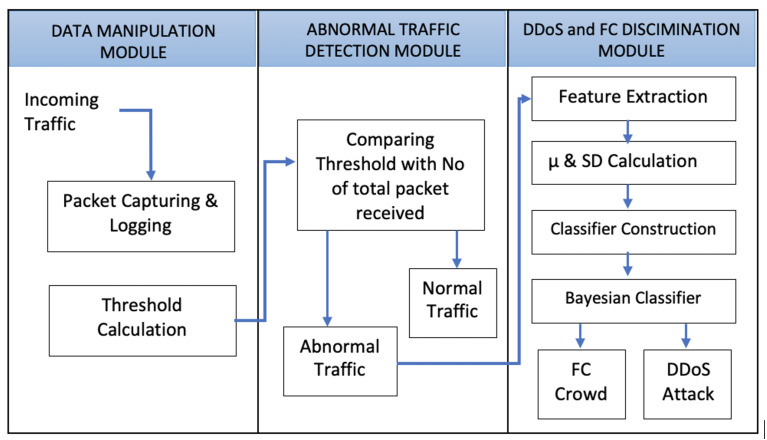
The proposed system model.

In Bayesian statistical inference, prior probability is the likelihood of an event occurring before fresh data is obtained. Before an experiment, this is the best rational judgment of the probability of an outcome based on current knowledge. The likelihood of the sequences, as well as each pair of values in the series, is expressed by the prior probability term. As additional data or information becomes available, the prior probability of an event will be altered to produce a more accurate measure of a probable outcome. The posterior probability is calculated using Bayes’ theorem based on the updated probability. The posterior probability, in statistical terminology, is the likelihood of event A occurring after event B has occurred. The prior probabilities can be computed by taking some data generated from real-world scenarios or artificial experiments. These probabilities can be considered as a tool for real-world forecasting values; however, the size of data for calculating these probabilities also matters. The input data should be of an adequate size to cover most of the possibilities that happen in the real world. Figure 1 shows the entire procedure of the proposed work.

**Figure 2:**
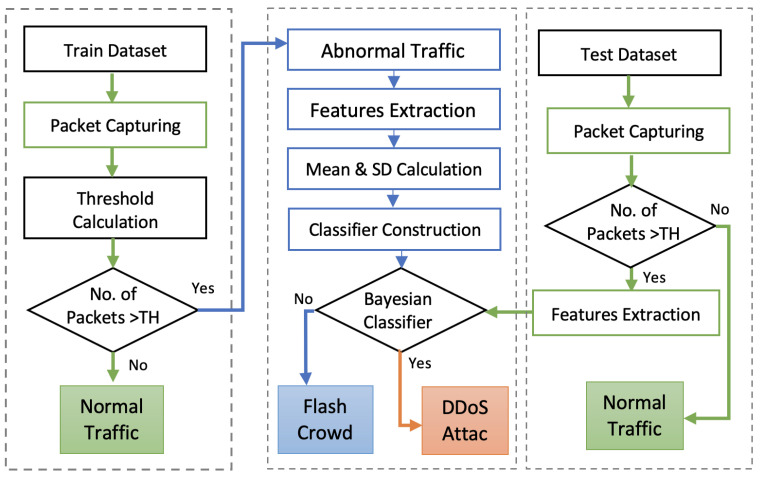
The overall structure of the proposed model.

The proposed mechanism is divided into four major segment processes. These are explained below as Section 3.1: Packets Capturing, Section 3.2: Traffic Categorization, Section 3.3: Feature Extraction, and Section 3.4: Traffic Classification. Figure 2 shows the structure of the proposed model.

The likelihood probability is the number of occurrences of each feature value for a precise attack or flash class value divided by the total number of instances belonging to that class. The posterior probability is the likelihood probability of an attack and normal class when the provided data formerly became part of an attack or flash class. Ultimately, the estimation is finalized by using the posterior probability. For computing, the posterior probability of the targeted cases, i.e., DDoS attack and FC classes, are considered. The posterior probability of a class instance is considered to belong in that particular class.

### 3.1. Packets Capturing

At the initial stage, the nodes are deployed in a network to form a WSN. These nodes constantly sense the data by investigating changes in the environmental parameters. These parameters can be air pressure, temperature, sound, heat, motion, etc. Once the data is collected, these nodes send their sensed data to BS through various routes. The data is sent in the form of packets. The sizes and number of packets depend on the size and nature of the data to be transmitted. A particular time interval is used between the transmission of data by each node. The BS captures the data in a time period that makes it capable of not losing any packet transmitted by the network nodes. The data packets received by the BS possess various properties such as the size, arrival time, source and route information, interpacket arrival time difference, etc. All these records are maintained by the BS for analysis.

### 3.2. Features Extraction

Once the data packets are captured by the BS, these are analyzed in the second stage. The packet contents, the data about the nature and features of packets, are recorded. The metadata about the packets is analyzed in this stage. The required parameters, i.e., IP addresses of the source and destination nodes, the size of the payload, and time of reception, are calculated and maintained by the BS.

### 3.3. Packets Categorization

The captured packets and the extracted features’ data are further categorized. The main categories are normal traffic and abnormal traffic. These categories are designed by taking some threshold values that are calculated by considering the dual median of the records. While designing an adaptive and intelligent system, it is obvious that the threshold value should not be a constant number. For this purpose, during the data capture, time slots, each having a minute, are made. Each time slot is further divided into twelve equal intervals. The median number of received packets in each interval is computed, as shown in Equation (1), where *n* is the number of packets in a particular time interval.

(1) $$median_{interval}=\frac{\left[n+1\right]}{2}th\;item$$

There are twelve intervals; therefore, twelve medians are obtained for each time slot of one minute. The same equation is applied again to calculate the median of medians. The value obtained from the median of the median is considered as the threshold value TH, as shown in Equation (2). In this case, mean and mode values cannot be considered. Because these values are highly susceptible when the data values have abnormal variations, however, in the case of normal data distribution, mean, mode, and median may give similar results and may be used interchangeably.

(2) $$TH=median_{slot}\left[median_{interval}\right]$$

In any time slot, if the number of packets is greater than the TH value, then it is considered suspicious traffic.

### 3.4. Traffic Classification

The detected suspicious traffic can be either an attack or an FC. In both cases, DDoS attack and FC, the mechanism detects abnormal traffic. Therefore, it is important to identify the nature of abnormal traffic. The FC traffic and DDoS attacks have a somewhat similar nature and disturb the network functionalities. However, the duration of FC might be shorter in many cases. These two anomalies have their own intentions and must be responded to separately. To classify these two types of anomalies, the Bayesian model is used. In this model, the variance in the sizes of payload and the packet reception time variance is considered. In the case of a DDoS attack, the time of each packet and its payload are important, as these keep some detectable traces. In a DDoS attack, these values are usually constant or equal to zero. However, in the case of FC, these values are random, or keep high variations.

Using the Bayesian model, the posterior probabilities of both classes of abnormal traffic are calculated. Each packet having the maximum posterior probability belongs to its class. When the traffic is detected as FC, the flash nodes are not blocked by the BS. The FC nodes, once identified, can access the network services continuously without any intervention, while in the case of a detected DDoS attack, the mechanism can block the involved nodes by taking their IP addresses into the block list. Some key operations in the proposed mechanism are explained as follows:

(a) First, two different flows are captured at the BS node. The receiving time and payload sizes are considered, and arithmetic mean μ and standard deviation σ are calculated, as shown in Equations (3) and (4).
  (3) $$\mu =\sum_{i=1}^{n}\frac{h_{i}}{n}$$
  (4) $$\sigma =\sqrt{\sum_{i=1}^{n}\frac{{\left(h_{i-}\mu \right)}^{2}}{n}}$$
(b) After obtaining these values for two flows, the standard deviations of both are checked. The flow with a higher standard deviation should be considered as a flash flow, and the other one is marked as an attack flow.
(c) Prior probabilities of both flows are computed by using Equations (5) and (6). In this equation, *a* denotes the number of packets class in the attack class, while fc is the total number of packets in the FC class.
  (5) $$p\left[a\right]=\left[\frac{a}{fc+a}\right]$$
  (6) $$p\left[fc\right]=\left[\frac{fc}{fc+a}\right]$$
(d) Equations (7) and (8) are used to calculate the likelihood probability of a packet, whether an attack or FC. This is based on the Gaussian probability density function. By using this equation, the probabilities for the size of payload and reception time are calculated.
  (7) $$p^{\prime }\left[\frac{h}{a}\right]=\frac{1}{\sqrt{2\pi \sigma_{ha}^{2}}}\times e^{-}\frac{{\left(hx-\mu \right)}^{2}}{2\sigma_{ha}^{2}}$$
  (8) $$p^{\prime }\left[\frac{h}{fc}\right]=\frac{1}{\sqrt{2\pi \sigma_{hf}^{2}}}\times e^{-}\frac{{\left(hx-\mu \right)}^{2}}{2\sigma_{hf}^{2}}$$
(e) The packet’s likelihood probability is obtained by taking multiplication of all the computed likelihood probabilities in Equations (9) and (10).
  (9) $$p\left[\frac{h}{a}\right]=\prod_{i=1}^{n}pi^{\prime }\left[\frac{h_{i}}{a}\right]$$
  (10) $$p\left[\frac{h}{fc}\right]=\prod_{i=1}^{n}pi^{\prime }\left[\frac{h_{i}}{fc}\right]$$
(f) Finally, the posterior probabilities of both the classes, the DDoS attack and FC, are computed by using Bayes theorem in Equations (8) and (9).
  (11) $$p\left[\frac{fc}{h}\right]=\frac{p\left[\frac{h}{fc}\right]\times p\left[fc\right]}{p\left[\frac{h}{fc}\right]\times p\left[fc\right]+p\left[\frac{h}{a}\right]\times p\left[a\right]}$$
  (12) $$p\left[\frac{a}{h}\right]=\frac{p\left[\frac{h}{a}\right]\times p\left[a\right]}{p\left[\frac{h}{fc}\right]\times p\left[fc\right]+p\left[\frac{h}{a}\right]\times p\left[a\right]}$$
(g) In Equation (9), the computed posterior probabilities of both attack and FC are compared to judge whether the receiving packets are associated with an attack or FC.
  (13) $$p\left[\frac{a}{h}\right]>p\left[\frac{f}{h}\right]$$
(h) In the class with a higher posterior probability, the packet is assumed to belong in that class.

The procedure is explained by using Algorithms 1 and 2.
**Algorithm 1** Training**Require:** Train Dataset**Ensure:** Normal, DDoS, FC  1: Begin  2: Incoming Known Traffic;  3: **for**
(i=1;i<Dataset.Length;i++)
**do**  4:  RL[i].IP⟵Pk.IP // Assignment to receiving list  5:  RL[i].RT⟵Pk.RT // RT-receiving time  6:  RL[i].PLS⟵Pk.PLS // Pls-payload size  7:  RL[i].P⟵Pk //Packet assigned  8: **end for**  9: **for** each *i* minute in RL.RT
**do** 10:  $m\left(interval\right).\left[i\right]⟵Med\left(\frac{No_{pkts}}{5secin1minute}\right)$ 11: **end for** 12: m(slot)⟵Med(m(interval)) // Median of all intervals 13: TH⟵m(slot) // Setting Threshold value 14: **if**
(Pkts<TH)
**then** 15:  Return “Normal Flow” 16: **else** 17:  **for**
(i=1;i<2;i++)
**do** 18:   $\mu F_{i}PM⟵\sum_{i=1}^{n}\frac{pkts[PLS_{i}]}{n}$ // mean calculation 19:   $\sigma F_{i}PS⟵\sqrt{\sum_{i=1}^{n}\frac{{\left(PLS_{i}-\mu PLS\right)}^{2}}{n}}$ // SD calculation 20:   $\mu F_{i}RM⟵\sum_{i=1}^{n}\frac{RT_{i}}{n}$ 21:   $\sigma F_{i}RS⟵\sqrt{\sum_{i=1}^{n}\frac{{\left(RT_{i}-\mu RT\right)}^{2}}{n}}$ 22:  **end for** 23: **end if** 24: $class_{i}⟵P\left(\frac{instanceofclass_{i}}{totalinstances}\right)$ 25: $p^{\prime }\left[\frac{h}{a}\right]=\frac{1}{\sqrt{2\pi \sigma_{ha}^{2}}}\times e^{-}\frac{{\left(hx-\mu \right)}^{2}}{2\sigma_{ha}^{2}}$ 26:  $p^{\prime }\left[\frac{h}{a}\right]=\frac{1}{\sqrt{2\pi \sigma_{hf}^{2}}}\times e^{-}\frac{{\left(hx-\mu \right)}^{2}}{2\sigma_{hf}^{2}}$ 27: End

Algorithm 1 takes the train dataset for training the system. This algorithm takes the records from the datasets by using a simple loop without any nested or complicated pattern. Therefore, it has time complexity with O(n). Furthermore, it can also be said that this algorithm has a linear time complexity because the running time is dependent upon the number of entries in the dataset. In this algorithm, up to step 8, the parameter values are taken from the inputted dataset. The loop in step 8 calculates the median of the number of packets for each minute. These are referred to as the medians of intervals. Step 12 calculates the median of all these computed medians. The ultimate median is considered as the threshold value at step 13. As per step 14, if the number of packets is less than the computed threshold value, it is considered as the normal flow. Otherwise, the means and standard deviations are calculated from step 17 to step 22. From step 24 to step 26, the classes are defined with the calculation of respective probabilities.
**Algorithm 2** Testing**Require:** Test Dataset**Ensure:** Normal, DDoS, FC  1: Begin  2: Incoming Unknown Traffic;  3: **if**
(Pkts<TH)
**then**  4:  Return “Normal Flow”  5: **else**  6:  **for**
(i=1;i<Dataset.Length;i++)
**do**  7:   RL[i].IP⟵Pi.IP  8:   RL[i].RT⟵Pi.RT  9:   RL[i].PLS⟵Pi.PLS 10:   Apply Classifier 11:   $p\left[\frac{a}{h}\right]=\frac{p\left[\frac{h}{a}\right]\times p\left[a\right]}{p\left[\frac{h}{fc}\right]\times p\left[fc\right]+p\left[\frac{h}{a}\right]\times p\left[a\right]}$ 12:   $p\left[\frac{fc}{h}\right]=\frac{p\left[\frac{h}{fc}\right]\times p\left[fc\right]}{p\left[\frac{h}{fc}\right]\times p\left[fc\right]+p\left[\frac{h}{a}\right]\times p\left[a\right]}$ 13:   **if**
$\left(p\left[\frac{a}{h}\right]>p\left[\frac{f}{h}\right]\right)$
**then** 14:    Return “DDoS” 15:   **else** 16:    Return "FC" 17:   **end if** 18:  **end for** 19: **end if** 20: End

Algorithm 2 is used for test data. This algorithm has a loop, but statements are executed according to the number of records. Therefore, it has O(n) asymptotic time complexity. Some statements of this algorithm are similar to the previous one. The abnormal traffic is handled at step 5. The parameter values are taken at steps 7, 8, and 9. The probabilities are calculated at steps 11 and 12. As per the calculated probabilities, the DDoS or FC traffic are distinguished.

## 4. Simulation Results

The proposed work is simulated in NS2 [26], under Red Hat 7. AODV protocol [27] is taken as a base protocol. CAIDA—DDoS Attack 2007 and FIFA World Cup are two real-world datasets used in this research. The CAIDA dataset contains around an hour of anonymized traffic traces from a DDoS assault that occurred on 4 August 2007. This form of denial-of-service attack tries to prevent users from accessing the targeted server by using all of the server’s computational resources and all of the bandwidth on the network linking the server to the Internet. To represent flash crowds, the World Cup 1998 dataset is used. The World Cup 1998 data is a compilation of requests made to www.france98.com (accessed on 3 March 2021) during the football tournament. In related studies, the World Cup 1998 dataset is used as a flash crowd dataset. While having these two datasets experimented with, it is assumed that the proposed mechanism will also be similarly effective with other types of real-world scenarios. There is no specific DDoS or FC related dataset seen that is meant for WSNs only. Moreover, the chosen datasets are mainly processed on the BS that has adequate resources and capable to perform similar to the nodes in the wired networks.

Additionally, various methods are utilized to create the dataset. Some fragments of the dataset are created from simulations and then from the real traffic to train and test the proposed system. The distribution of nodes in the dataset is designed as follows: (a) attack nodes are 30%, (b) normal nodes are 40%, and (c) FC vectors are 30%. This section is further divided as follows: In Section 4.1, calculation of threshold value is discussed. The classification of normal and abnormal nodes is performed in the first experiment discussed under Section 4.2. In Section 4.3, the abnormal nodes’ traffic is further analyzed to determine the attack and flash traffic flows. In Section 4.4 and Section 4.5, the same procedure is repeated to obtain another set of results. The experimental results on the real dataset are given in Section 5. The key parameters are listed in Table 2.

### 4.1. Threshold Value

Different experiments were performed to obtain the threshold value for varying numbers of nodes (30, 40, 50, 100, and 200). However, we obtained the optimal result for 30 nodes. For threshold value, 30 out of 100 nodes were selected to transmit packets with different data rates varying from 10 kbps to 40 kbps. The total simulation time was set to 60 s, during which the nodes transmitted their data towards the BS. The simulation time was then 12 intervals each having a slot of 5 s. According to the procedure, the median of the received packets was computed for each interval. A total of 12 median values were computed for the entire simulation time. Another median was computed of these obtained 12 median values. The ultimate value computed was 167 vectors per slot. This value is considered as the threshold value.

### 4.2. First Experiment

**Figure 3:**
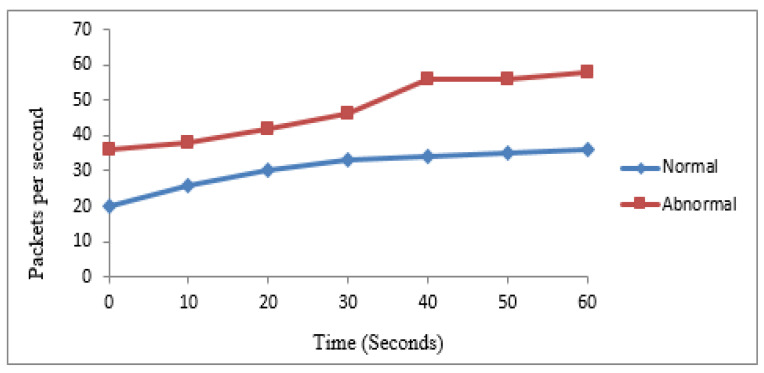
Experiment 1 for categorization of traffic.

The first experiment was performed based on 30 nodes to verify the performance of the mechanism for abnormal and normal traffic categorization. According to the procedure, 10 nodes were identified as normal while the rest were abnormal. Table 3 shows the number of packets generated by normal and abnormal nodes in 60 s. The same values are graphically represented in Figure 3.

### 4.3. Classification of Traffic

**Figure 4:**
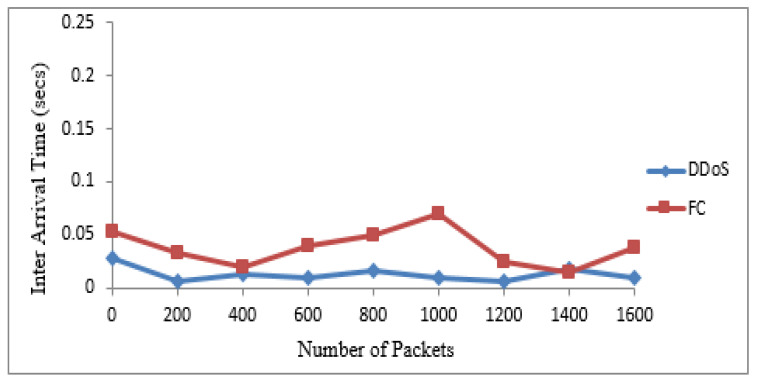
Discrimination of DDoS attack from FC based on IAT.

**Figure 5:**
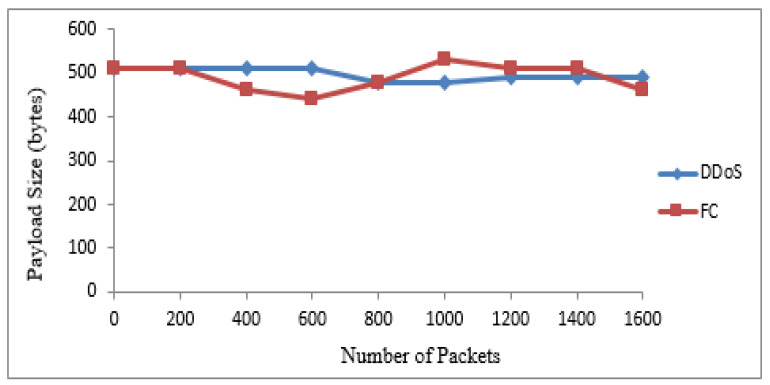
Discrimination of DDoS attack from FC based on the size of payload.

The 20 suspicious nodes were further classified as attacker and flash nodes. The attacker nodes frequently transmitted packets from 20 to 40 kbps with the same sized packets with a constant timespan, while flash nodes frequently transmitted random-sized packets with an irregular time span from 25 kbps to 45 kbps. The means and standard deviations of receiving time and sizes of payload of both types of traffic were calculated. The class with maximum standard deviation was labeled as FC, while the other one was DDoS attack. The Bayesian model takes these means and standard deviations for classification of irregular traffic further in attack and FC traffic. The proposed system identified that 14 nodes are flash and 6 are attacker nodes in 20 suspicious nodes. Table 4 and Table 5 show the discrimination of DDoS attack from FC traffic computed from interarrival time variance and size of payload variance. The same are also represented by Figure 4 and Figure 5. The dataset used for these two types of traffic is shown in Table 6.

### 4.4. Second Experiment

**Figure 6:**
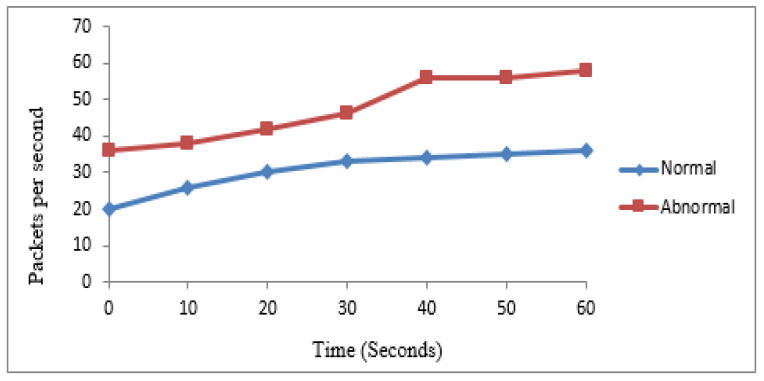
Experiment 2 for categorization of traffic.

A second experiment was performed based on 40 nodes to evaluate the proposed mechanism’s performance for abnormal and normal traffic categorization. The proposed mechanism identified 15 as normal and 25 as suspicious nodes. Figure 6 shows the categorization of traffic in normal and abnormal traffic.

### 4.5. Classification of Traffic

**Figure 7:**
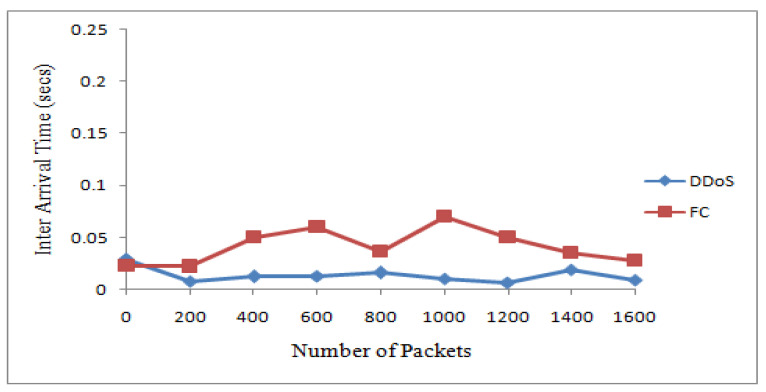
Discrimination of DDoS attack from FC based on IAT.

**Figure 8:**
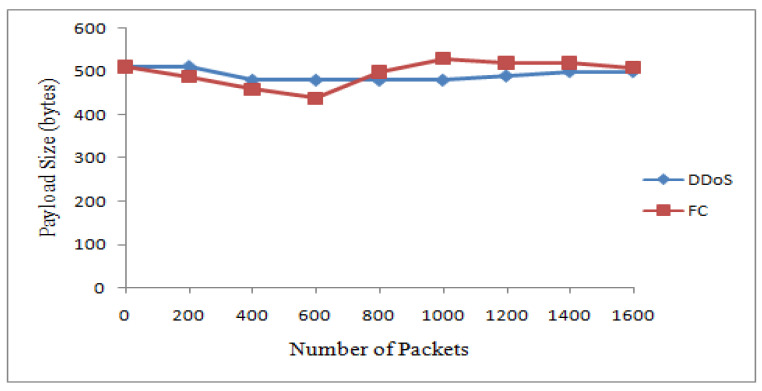
Discrimination based on the size of payload.

The suspicious nodes were further classified as attacker and flash nodes. Flash nodes have data rates of 20 kbps to 40 kbps and attacker nodes’data rates vary from 25 kbps to 45 kbps. Means and standard deviations of payload size and receiving time of payload size were calculated. The class with maximum standard deviation was labeled as FC while the other one was DDoS attack. The Bayesian model takes these means and standard deviations for classification. The proposed mechanism identified 15 nodes as flash and 10 as attacker nodes. Table 7 shows the dataset for these two types of traffic. The graphs in Figure 7 and Figure 8 show the classification of traffic based on interarrival time variance and payload size variance.

## 5. Simulation Results by Using Real Datasets

For further evaluation, two real-world datasets were also used. These datasets are “1998 FIFA World-Cup” [28] for FC data and “CAIDA DDoS Attack 2007” [29] as DDoS data. From the CAIDA dataset, one minute (4 August 2007 from 05:30 to 05:31) of traffic data was selected. From the FIFA World cup dataset, one hour (10:06:1998; 15:00 to 16:00) was selected. In one hour of the CAIDA attack dataset, 359,655,826 request packets were generated, while in one minute, this value was 296,361. Similarly, in the FIFA World Cup, 253,830 request packets were generated in one hour. In this dataset, the requests are produced from legitimate web clients to the FIFA website. Table 8 shows the computed parameters in this experiment.

**Figure 9:**
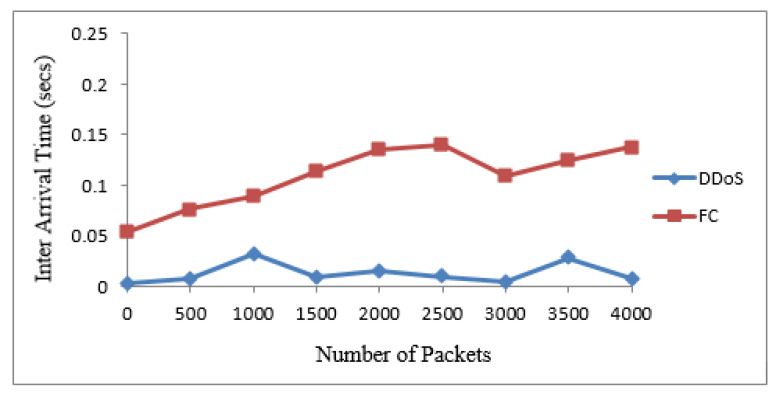
Discrimination of DDoS attack from FC based on IAT.

**Figure 10:**
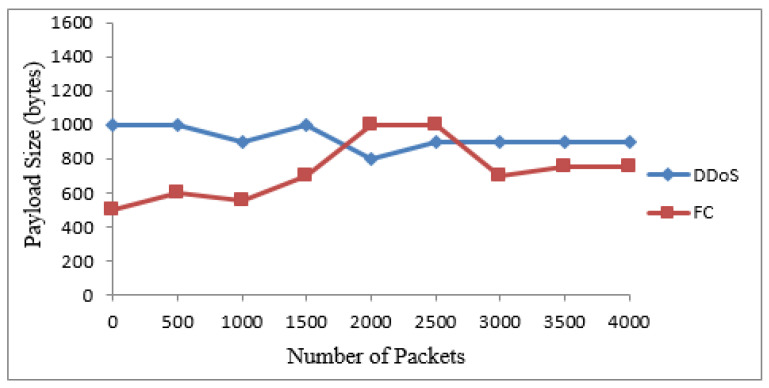
Discrimination of DDoS attack from FC based on the size of payload.

Figure 9 and Figure 10 show the results for traffic classes from the two real datasets. It is noted that the packets are almost the same sizes as in the case of the attack packets. In addition, the arrival time for such packets is less. The dataset records show that the attack packets are merely produced and transmitted. These data packets do not carry data and do not need any time for transmission. In the case of the FIFA World Cup dataset, the generated packets are of different sizes and their interarrival times are also not in the same pattern.

**Figure 11:**
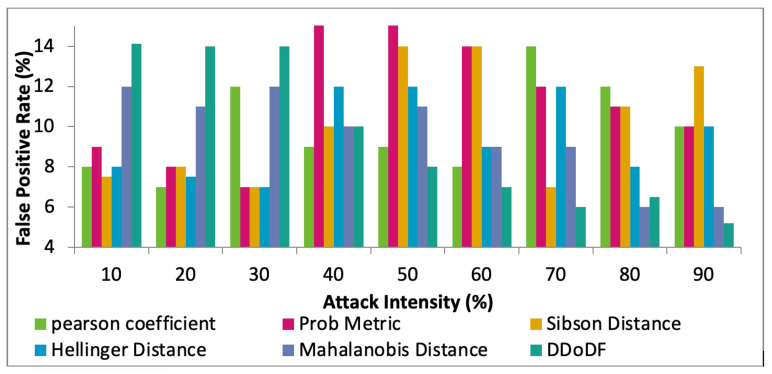
Attack intensity vs. false-positive rates.

**Figure 12:**
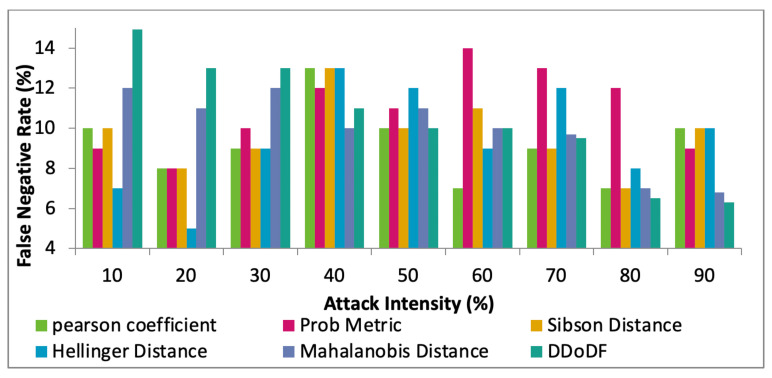
Attack intensity vs. false-negative rates.

**Figure 13:**
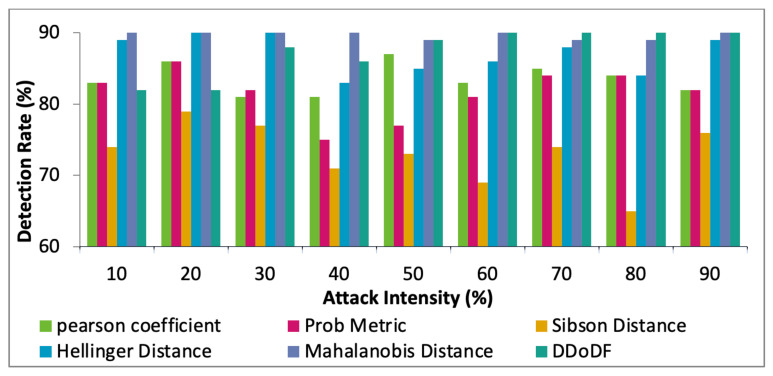
Attack intensity vs. detection accuracy.

Furthermore, the performance of the proposed DDoDF is compared with the existing mechanisms, Hellinger distance [8], Sibson distance [19], probability metric [20], Pearson coefficient [21], and Mahalanobis distance [24], while having two real datasets taken. The intensity of attack differs from 10% to 90%. Figure 11, Figure 12 and Figure 13 show the comparative results of the selected mechanisms with our mechanism. Varying attack intensities are taken. The attack intensity is measured based on the data traffic rate. The normal traffic rate is compared with the suspected abnormal traffic. If both are equal, then the attack intensity is equal to 0%. The highest known difference between the two traffic rates is assumed to be 100%.

The key terms calculated are false-positive rate (FPR), false-negative rate (FNR), and rate of detection. In the beginning, DDoDF has lower values having higher false positives and false negatives. With the passage of time, the proposed mechanism evolves and learns from the data traffic based on the likelihood probability associated with receiving time and payload size. Once the system is trained, it is fully configured and loaded to obtain a high detection rate with low false-positive and false-negative rates.

Figure 11 shows the FPR against the varying attack intensities. The results show that the proposed protocol has the highest value, i.e., 14.93%, when the attack intensity is at 10%. However, as the intensity is increasing, the FPR value is inversely reduced. At 90% of attack intensity, the value for FRP is 5.2%, which is the least among the competing protocols. Mahalanobis distance has a somewhat similar performance in this test. The proposed mechanism operates mainly on the probabilities. The naïve-based probabilistic model performs much better with higher abnormal traffic, causing less values for FPR.

Figure 12 shows the FNR against the attack intensities starting from 10% to 90%. Initially, with the low level of attack intensity, the proposed model does not perform well if compared with other mechanisms. Initially, it gives 14.12% for 10% attack intensity. However, the values for FNR drastically decline with higher attack intensities. For 90% attack intensity, DDoDF gives 6.3% FNR, which is the lowest value among all the test mechanisms. The main reason is similar to the previous case of FPR.

In Figure 13, the considered mechanisms are tested for detection rates with varying attack intensities. With 10% attack intensity, Hellinger distance and Mahalanobis distance give much better results. The proposed DDoDF gives moderate values in the beginning. However, as the attack packets are increased, the detection rate of DDoDF is also increased. After 40% attack intensity, the DDoDF gives a slightly better detection rate than Hellinger distance and Mahalanobis distance. The proposed work has multiple levels for the detection and identification of abnormal traffic. Therefore, it requires an adequate level of attack traffic for better performance.

## 6. Conclusions and Future Work

In this research work, a novel detection and discrimination mechanism is introduced to detect the abnormal traffic and discriminate DDoS attacks from FC. Both types of traffic have common features in general; however, some key characteristics are different, and can be used to distinguish them from each other. In this mechanism, various steps are followed to obtain the target objective. The traffic is analyzed, and the analysis data is further processed. The key parameters considered are the number of packets, size of the payload, and interpacket arrival time variances. Ultimately, a naïve Bayesian model is used to detect FC traffic and separate it from the DDoS attacks. Various simulations are made to verify the performance of the system and are also compared with some existing techniques. Our experimental and simulation results show that the proposed detection system can differentiate DDoS attack traffic from FC with more than 93% accuracy, with 5.2 FPR and 6.3 FNR based on the two real datasets (1998 FIFA World-Cup and CAIDA DDoS attack 2007). In the initial tests with the low level of attack intensity, the proposed work does not perform well. With 10% attack intensity, it gives 14.12% FPR and 14.9%3 FNR, which are not good among the competitors. This is because it operates on the naïve-based probabilistic model with multilevel probabilities. Such types of models require an adequate amount of data so that optimal and more accurate results are achieved. However, for attack intensities of 40% to 90%, the proposed mechanism outperforms all the mechanisms. For the highest attack intensity, DDoDF gives around 93% detection rate, 5% FPR, and 6% FNR, which are the most optimal values in the experiments. The results are encouraging and prove that the proposed mechanism performs well in the target domain.

In the future, the detection accuracy can be enhanced by combining some additional parameters, such as payload patterns and hop count information, with the existing parameters to build a strong detection system. The proposed system can also be simulated on multiple datasets, i.e., HTTP logs, NLANR, and MIT Lincoln, with additional parameters for evaluation. This work can be implemented on other ad hoc networks with some alterations. This work only detects and discriminates DDoS attack traffic from FC, and it does not prevent the network from DDoS attacks. The prevention module can also be added to the detection module to prevent all the attacker nodes in the network.

## Figures and Tables

**Table 1:** Summary of related articles.

Article	Details
Katiyar et al. [18]	Parameters: IP and Port addresses of Source & Destination Validation Technique: Simulation Dataset: No dataset used Detection Metrics: Entropy variation Limitations: When the traffic increases the performance degrades low efficiency
Yu. et al. [22]	Parameters: Source IPs distribution, access intent, traffic rate Validation Technique: Simulation Dataset: DDoS⟶MITLincoln&FC⟶HTTPlogs Detection Metrics: Correlation coefficient total variation Limitations: Degrade for high-rate DDoS attack Only flow similarity is not suitable
Bathia et al. [11]	Parameters:Variation in the source addresses and traffic rate, packets scattering among source addresses Validation Technique: Real-time Dataset: DDoS⟶CAIDA2007&FC⟶1998FIFAworld−cup Detection Metrics: Statistical calculation Limitations: Not have any accuracy
S.Renukadevi et al. [8]	Parameters: Flow similarity, client legitimacy, page referred Validation Technique: Simulation Dataset: DDoS⟶CAIDA2007&FC⟶1998FIFAworld−cup Detection Metrics: Hellinger distance Limitations: Accuracy is 91%. Environment specific
J. Gera et al. [23]	Parameters: Source entropy & traffic entropy Validation Technique: Simulation Dataset: No Dataset Used Detection Metrics: Entropy
K. S. Sahoo et al. [25]	Parameters: Source & destination IPs, source & destination port Validation Technique: Simulation Dataset: DDoS and FC datasets generate through Scapy tool Detection Metrics: General entropy & generalized information distance
S. Daneshgadeh et al. [24]	Parameters: Time interval, source/destination IPs Validation Technique: Simulation Dataset: CAIDA 2007 for DDoS attack & 1998 FIFA World Cup for FC Detection Metrics: Shannon entropy, Mahalanobis distance, kernel online anomaly detection
Wang et al. 2017 [15]	Parameters: Structural, such as temperature, humidity, light intensity, and voltage; Validation technique: Simulation; Dataset: IBRL dataset with manual entries; Target: Detection of abnormal structural events
Reddy and Thilagam, 2020 [16]	Parameters: Packet size, port number, source address, destination address, and jitter; Validation Technique: Simulation; Dataset: None; Target: DDoS detection and mitigation

**Table 2:** Parameters for experimental simulation.

Parameter	Values
Simulator	NS-2.33
Duration of Simulation	60 s
Nodes’ Transmission range	250 m
Network Area	1000 × 1000 m
Base Protocol	AODV
Number of Nodes	100–200
Nodes’ Distribution	Random
Traffic source	CBR
Maximum speed of node	10 m per second
Packet Size	Random
Nodes’ Pause Times	10 to 60 s

**Table 3:** Comparison of traffic.

Traffic Type	Number of Packets per Second					
Time (s)	10	20	30	40	50	60
Normal	24	26	32	34	36	38
Abnormal	37	38	48	58	64	64

**Table 4:** DDoS attack versus FC based on IAT.

Traffic Type	Interarrival Time							
Received Packets	200	400	600	800	1000	1200	1400	1600
DDoS	0.00743	0.01135	0.01283	0.01600	0.01010	0.00643	0.01923	0.00889
FC	0.02215	0.05002	0.05999	0.03656	0.07009	0.04996	0.03533	0.02778

**Table 5:** DDoS attack from FC based on the size of payload.

Traffic Type	Payload Size (Bytes)							
Received Packets	200	400	600	800	1000	1200	1400	1600
DDoS	512	512	512	480	480	490	490	490
FC	512	460	440	480	530	510	512	460

**Table 6:** DDoS and FC traffic classification.

Type	IPs	σ(PLsSize)	σ(Time)	μ(PLsSize)	μ(Time)
FC	14	18.1818	0.0285	490.33	0.0472
DDoS	6	10.0484	0.00780	500.83	0.00742

**Table 7:** Parameters calculated for attack and FC.

Type	IPs	σ(PLsSize)	σ(Time)	μ(PLsSize)	μ(Time)
FC	15	23.214	0.02973	483.33	0.037
DDoS	10	5.0484	0.00130	503.12	0.00432

**Table 8:** Parameters calculated for DDoS attack and FC traffic.

Type	IPs	σ(PLsSize)	σ(Time)	μ(PLsSize)	μ(Time)
FIFA World Cup	8106	6130.3	0.3498	10,055.1	27.8181
CAIDA DDoS	5556	0.90	0.001123	60.8	0.0048

## Data Availability

Not applicable.

## List of Symbols

Symbol	Description
*n*	Number of packets
*TH*	The threshold value of packets
μ	Mean of a packet
µFiPM	Flow fi payload mean
FiPS	Flow fi payload standard deviation
*µFiRM*	Flow fi receiving time mean
FiRS	Flow fi receiving time standard deviation
$h_{x}$	Attribute x
*M*	Network
$p[h{\left[/f\right]}_{c}]$	Likelihod probability of flash crowd
p[h/a]	Likelihood probability of attack class
*RL*	Receiving list
*interval*	Period between two nodes
*slot*	Combination of intervals
Pi	Packets
σ	Standard deviation
p[f_(c)]	Prior probability of flash crowd
*p[a]*	Prior probability of attack class
p $\left[f_{c}/h\right]$	Posterior probability of flash crowd
p[a/h]	Posterior probability of DDoS attack
*n*	Total number of nodes
$class_{i}$	Prior probability of class
PLS	Payload size of packet
*RT*	Received time of packet
